# Reliability of voluntary step execution behavior under single and dual task conditions

**DOI:** 10.1186/1743-0003-4-16

**Published:** 2007-05-29

**Authors:** Itshak Melzer, Irena Shtilman, Noah Rosenblatt, Lars IE Oddsson

**Affiliations:** 1 NeuroMuscular Research Center, Boston University, Boston, USA; 2 Sargent College of Health & Rehabilitation Sciences, Boston University, Boston, USA; 3 Dept Phys Therapy, Faculty of Health Sciences, Ben-Gurion University of the Negev, Beer-Sheva, Israel; 4 Sister Kenny Rehabilitation Institute, Sister Kenny Research Center (12101), 800 E. 28th St. Minneapolis, MN 55407, USA

## Abstract

**Background:**

The current study investigated the repeatability (test-retest reliability) of ground reaction force parameters recorded during a voluntary step execution under single (motor task) and dual task (motor and cognitive task) conditions for healthy adults and elderly individuals as well as the number of trials required to produce repeatable results.

**Methods:**

Twenty-four healthy adults (21–63 years old) and 16 elderly adults (66–87 years) performed a voluntary rapid step execution following a tap on their heel while standing on a force platform under single and dual task conditions on three separate occasions. The first two tests were performed 30–60 minutes apart and the third test was performed a week later. Variables analyzed from the ground reaction force data included onset latency of step initiation (initiation phase), preparation and swing phases, foot-off and foot-contact times.

**Results:**

Intraclass correlation coefficients (ICC(2,1)) were good to excellent across all parameters and test conditions for the pooled population and for elderly (0.74–0.92 and 0.62–0.88, respectively) except for the swing phase duration where lower values were seen (0.54–0.60 and 0.32–0.64 respectively). Values were similar under single and dual task conditions.

**Conclusion:**

A voluntary step execution test, performed under single and dual task conditions especially foot-off and foot-contact times, is a reliable outcome measure that may be a useful tool to asses dynamic balance function for diagnostic purposes as well as clinical intervention trials.

## Background

Postural control plays a fundamental role for our ability to maintain balance during various activities of daily living especially those that include elements of independent standing and gait. Age-related deterioration of the postural control system can lead to balance impairment and limitations of mobility causing disability that may contribute to falls. Falls are the leading cause of injury-related visits to emergency departments and the primary etiology of accidental deaths in persons over the age of 65 years [1-4]. Nearly 30% of elderly individuals over 65 and almost 50% of elderly individuals over 80 fall at least once every year [3]. Various postural responses including rapid execution of a step may prevent a fall from occurring [5]. Protective stepping [6], a perturbation-triggered automatic response that is not under direct volitional control, can quickly increase the base of support to help maintain equilibrium [6,7]. In addition, rapid voluntary stepping can help prevent the occurrence of a fall [8], especially under circumstances when there is no distinct postural perturbation but rather a gradual change in posture. Slow postural changes could occur during various activities of daily living such as walking, rising from a chair, tripping or tangling of the feet, as well as during reaching movements, stumbling on a carpet, rug or inappropriately placed furniture or cords, circumstances under which the majority of falls occur in the elderly population [9,10]. The faster a step is executed in these situations the lower the risk of a fall would be thereby providing a rationale for using measures of rapid voluntary step execution as a potential indicator of the ability to avoid falls [8].

In a real life situation, the need to rapidly step to prevent a fall would likely occur when attention is not directly focused on performing a motor task but rather on a cognitive task such as reading a street sign or watching traffic. Simultaneous performance of cognitive and postural tasks has been suggested as a potential contributor to instability and falls [11,12] and there is evidence that attention-demanding tasks have an effect on postural control in aging [13]. Therefore, it seems reasonable to hypothesize that falls are not due to balance deficits in isolation, but to the inability to effectively allocate attention to balance under multi-task conditions [14,15] possibly due to poor executive function, commonly seen in elderly subjects [16]. Further support for this view is provided by results showing more than a doubling of time to initiate a voluntary step under dual as compared to single task conditions in healthy elderly subjects following a cutaneous tap stimulus compared to a 34% increase in young subjects [8]. During a voluntary step execution test the duration of various temporal phases extracted from the ground reaction force can provide information regarding executive function (Initiation Phase – time from stimulus to beginning of mediolateral weights shift prior to any movement), associated postural control in preparation for the step (Preparation Phase – time from Initiation Phase to foot-off) and muscle power output (Swing Phase – time from foot-off to foot contact) [8]. The sum of these three phases is equal to the foot contact time, the overall time to execute the step following the initial cutaneous cue. From a clinical view the foot contact time as a parameter may provide important information regarding an individual's ability to resist a fall in a given situation, whereas the different phase durations may indicate specific deficiencies in the performance profile of the step.

The aim of the present study was to investigate inter- and intratester reliability of the temporal parameters assessed during a Voluntary Step Execution Test [8] under single and dual task conditions, i.e. simultaneous performance of a motor and a cognitive task. We also investigated the number of trials required to obtain repeatable results. If step execution time is related to fall risk, this information may be of use in a clinical or laboratory setting to assess step execution performance or to evaluate effectiveness of rehabilitation of postural control and balance function.

## Methods

### Subjects and procedure

Twenty-four healthy adult subjects (mean, 36.5 ± 15.5, range, 21–63 years old) and sixteen healthy elderly subjects (mean, 77.6 ± 6.6 years, range 66–87 years old) were recruited for the study. Elderly subjects were included based on the following criteria: no previous neurological or orthopedic disorders, a score greater than 45 on the Berg Balance Scale [17], a Mini-Mental Score [18] greater than 24 indicating the absence of moderate to severe dementia, absence of serious visual impairment or color blindness, and the ability to ambulate independently (use of cane allowed but not walker). Elderly subjects without balance impairment were chosen since age-related deterioration of balance function, affects all elderly individuals [19]. This may lead to an increased risk of falling even in healthy elderly persons, therefore, a better way to evaluate and than decrease the number of fall-related injuries in the elderly may be to also direct preventive efforts towards elderly individuals who have not yet fallen.

All subjects provided informed consent, in accordance with approved procedures by the Boston University Charles River Campus IRB and by the Helsinki ethics committee at Soroka Medical Center and Ben-Gurion University of the Negev, Israel. All subjects, apart from two subjects of the younger group, participated in two separate data collection sessions separated by one week. To assess intertester reliability, voluntary step execution behavior under single and dual task conditions were tested 30–60 minutes apart by two trained raters (Rater 1, or Rater 2) then by Rater 3. Raters were blinded to each others results. Also, intratester reliability of voluntary step execution behavior under single and dual task conditions was assessed by testing subjects one week apart, by the same raters (#1 and #2) testing the same subjects. The rater was blinded to results from the previous week and the results were not communicated to the subjects.

### Instrumentation and data analysis

**Figure 1:**
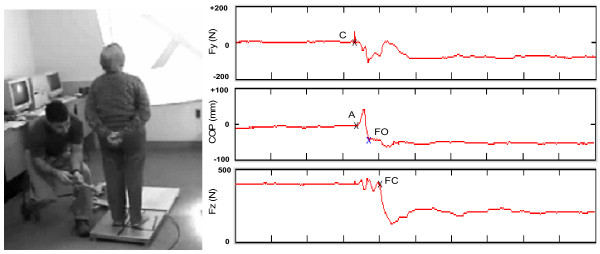
Photo of the experimental set-up showing an example of forward Step Execution Test being performed under during single task conditions (left). Example of step execution data (right). Fy = Ground reaction forces (shear forces) in antero-posterior direction. Fz = Vertical ground reaction forces, COPx = Mediolateral center of pressure, N = Newton, mm = Millimeter. The following events are marked with X; Tap cue (C); Initial deviation of COPx (A); Foot-off (FO); Foot contact (FC). Note that the tap cue is detectable in any of the signals. See text for further details.

A portable Kistler 9287 force platform was brought to the community dwelling center to measure center of pressure (COP) and ground reaction force data during each step execution trial. Force data were sampled at a frequency of 100 Hz and stored for later off-line analysis [8]. Subjects were instructed to adopt a standardized stance with their feet abducted 10 degrees and their heels separated mediolaterally by 6 cm [20,21]. The repeatability of foot position between trials was controlled through the use of a rigid template that forced the feet into the proper position at the start of each trial. The template was removed prior to the start of data collection. Stepping foot was the same for all trials and chosen by the subject. Subjects knew beforehand which foot to step with and in which direction to step. Three forward and three backward stepping trials were performed in a randomized order for each of the two task conditions. During the single task trials, subjects were asked by the rater to view an 'X' projected at eye level onto a wall 3 meter in front of the subject (Figure 1, left). Subjects were instructed to stand evenly on both feet and to step as quickly as possible following a distinct tap on the heel of the stepping foot provided manually by the rater using a 30 cm long foam-padded wood baton. Subjects were allowed to practice to become familiar with the test situation and to ensure step clearance of the force platform.

Following completion of the six single task trials, subjects repeated the protocol under dual task conditions performing a modified-Stroop test [22]. The modified-Stroop test consisted of a 5 by 5 matrix with names of colors where the color of the ink was always different from the name of the color. For example, the word "red" was printed in yellow ink. Subjects were asked to step as quickly as possible from the force plate while reading out loud the color of the ink of the projected color name. The modified Stroop-test was used because it requires focused attention and few instructions to perform. In addition, it requires only direct verbal responses and it does not address memory, which may be impaired in the elderly. After completion of all twelve trials (six single task and six dual task trials) with the Rater 1 and Rater 2 subjects were given a 30–60 minute rest period. Following rest, the protocol was repeated with Rater 3 to complete the first test session. Subjects returned within a week for the second test session and the test protocol was repeated by Rater 1.

Force platform data were analyzed using code written in Matlab (Math Works Inc, Cambridge, MA, USA) [8,23] to extract five different temporal parameters: step initiation, preparation and swing phases, foot-off time and foot contact time (Figure 1, right). The tap cue was detected as a spike (greater than three standard deviations from the average baseline noise) in the ground reaction forces in the anteroposterior direction. Step initiation was defined at the first mediolateral deviation of center of pressure (COP) towards the swing leg (greater than 4 mm from the average baseline sway prior to tap). The step initiation phase was calculated as the time from tap onset to step initiation. Foot-off time was defined by a sudden change in the slope of COP towards the stance foot in the mediolateral direction. Foot-contact time was defined as the onset of unloading in the vertical ground reaction forces [24]. The preparation phase was defined as the time from step initiation to foot off and swing phase was calculated as the time from foot-off to foot- contact [8]. Each of the five temporal parameters was determined for each of the twelve trials for both raters and sessions. An average of each event across all six trials during two different conditions: single task and dual task was used to represent each subject.

Shapiro-Wilk's statistic was used to test normality of the variables for pooled population and for both groups [25]. The effects of age and task condition on the mean dependent variables were calculated with SPSS (version 10.1, Chicago, IL) using a two-way repeated-measures analysis of variance (ANOVA) that included group (adults – elderly adults) as the between subjects factor with repeated measures on the within subjects factors of task (single – dual). Wilcoxon's signed rank test and Mann-Whitney U-tests were used in case the variable was not normally distributed. The dependent variables were; Step Initiation Phase duration; Time to Foot-off; Time to Foot-contact; Preparatory Phase and Swing Phase durations. A significance level of 0.05 was used.

Once the timing of the five temporal events was determined, inter- and intratester reliability was assessed using a two-way random model for intraclass correlation coefficient (ICC) [26]. We used ICC(2,1) which assumes each subject is assessed by each rater and the raters are randomly selected and reliability calculated from a single measurement. Intertester reliability compared the results of the first and second testers from the first session; additional intratester reliability test compared the results of the tests during the first and second session. Three populations were considered: elderly adults (N = 16), adults (N = 24) and a pooled population consisting of all subjects (N = 40). For each population, ICC(2,1) was determined for each of the five temporal events during each of the two test conditions, without regard for direction (overall stepping). The following guidelines were used when interpreting ICC magnitudes: ICC < 0.4 represents poor reliability, 0.4 ≤ ICC ≤ 0.75 represents fair to good reliability, and ICC > 0.75 represents excellent reliability [26]. Cronbach's alpha was calculated to estimate internal test consistency using reliability analysis procedures in SPSS.

## Results

Each subject performed 36 trials (12 trials × 3 sessions) 18 of which were under single (6 trials × 3 sessions) and 18 under dual task conditions (6 trials × 3 sessions). Two subjects of the younger adults group did not perform the third session due to illness. Table 1 shows average values of the temporal step parameters across all three test sessions for both elderly and younger adults under single and dual task conditions, all step parameters except for swing phase duration were normally distributed (Shapiro-Wilk's statistic not significant) for the pooled population and for each age group. In agreement with previous work [8] there were statistically significant differences between the younger group and elderly individuals across all step execution parameters for both task conditions (marked with * in Table 1). With the exception of the swing phase in the younger group, there was a statistically significant within group increase in all other parameters under the dual task condition (marked with + in Table 1).

**Table 1:** Mean of step execution parameters for younger and elderly adult subjects

	**Elderly Adults**	**Younger adults**
**Single task**		
**Initiation Phase**	205 ± 55 *	135 ± 25
**Preparation Phase**	420 ± 75 *	340 ± 62
**Swing phase**	371 ± 145 *	283 ± 48
**Foot off time**	625 ± 119 *	475 ± 78
**Foot contact time**	996 ± 223 *	758 ± 112
**Dual task**		
**Initiation Phase**	433 ± 158 *^+^	186 ± 51^+^
**Preparation Phase**	470 ± 105 *^+^	364 ± 50 ^+^
**Swing phase**	391 ± 144 *^+^	281 ± 61
**Foot off time**	902 ± 236 *^+^	550 ± 76 ^+^
**Foot contact time**	1294 ± 332 *^+^	831 ± 119 ^+^

Values shown represent averages from the 1^st ^measurement session in milliseconds for single (6 trials, 3 forward + 3 backward) and dual task (6 trials, 3 forward + 3 backward) ± 1 standard deviation. * Indicates statistically significant differences between age groups and ^+^between task conditions within age groups, respectively (p < 0.05). P-value compares swing phase means ( ± 1 Standard Deviation) between the two groups were measured using Wilcoxon test and Mann-Whitney U test.

Intertester reliability across all step parameter except for the swing phase ranged between 0.74–0.89, for Rater 1 vs. Rater 3 and between 0.74–0.84, for Rater 2 vs. Rater 3 under single task condition and 0.75–0.86 and 0.77–0.82 under dual task conditions, respectively. These were not significantly different which allowed pooling of the results for Rater 1 and Rater 2. Furthermore, ICC values across all step parameter except for the swing phase, between forward and backward stepping direction under either single or dual task conditions were similar (0.70–0.89 and 0.66–0.90, respectively) which allowed pooling of results across the two step directions.

### Intertester reliability

Table 2 shows ICC's for intertester reliability across both step directions for all temporal events during single and dual task conditions. Intertester ICC values for the pooled population (N = 40) were excellent (0.79–0.88, p < 0.0001) for both single and dual task stepping conditions with the exception of a somewhat lower value, yet statistically significant, for the swing phase under single and dual task conditions (ICC = 0.60, p < 0.0001 and ICC = 0.55, p < 0.03 respectively). ICC values for the elderly group were good to excellent for all parameters across both test conditions (0.70–0.83, p < 0.001, Table 2) with the exception of ICC for the swing phase under dual task conditions which was poor and not statistically significant (ICC = 0.32, p = 0.11, Table 2). Similarly, ICC values for the younger adult subjects were good to excellent (0.68–0.88, p < 0.000, Table 2) except for ICC's of the swing phase in single and dual stepping, which were fair to good and statistically significant (ICC = 0.46, p = 0.01 and ICC = 0.77, p < 0.000, respectively, Table 2).

**Table 2:** ICC values for intertester reliability

		**IPD**	**PPD**	**SPD**	**FOT**	**FCT**
**Single Task**	Elderly N = 16	0.79***	0.76***	0.64**	0.82***	0.82***
	Younger N = 24	0.70***	0.78***	0.46**	0.79***	0.78***
	Pooled N = 40	0.86***	0.81***	0.60***	0.87***	0.84***
**Dual Task**	Elderly N = 16	0.70***	0.70***	0.32 ns	0.83***	0.74***
	Younger N = 24	0.73***	0.88***	0.77***	0.68***	0.82***
	Pooled N = 40	0.79***	0.82***	0.55*	0.88***	0.86***

Table 2 shows ICC(2,1) values and significance levels (in parenthesis) for intertester reliability (Rater 1 vs. Rater 2) for step execution parameters Initiation Phase Duration (IPD), Preparatory Phase Duration (PPD), Swing Phase Duration (SPD), Foot-Off Time (FOT) and Foot-Contact Time (FCT) under single and dual task conditions calculated separately for elderly subjects (N = 16), younger adult subjects (N = 24) and the pooled population of elderly and younger adult subjects (N = 40). An average of all six step trials (three forward and three backward) was used in the analysis (ns – not significant, *p < 0.05, **p < 0.01, ***p < 0.001).

Foot-contact and foot-off times showed the highest ICC values for elderly persons under both single and dual task conditions (0.82–0.82, p < 0.000 and 0.74–0.83, p < 0.000 respectively). The highest values for internal test consistency were also found for foot-contact and foot off times (Cronbach's alpha, ranged between 0.85 and 0.91), whereas the lowest were for swing phase duration (Cronbach's alpha, between 0.48 and 0.78).

### Intratester reliability

ICC values for intratester reliability of the elderly, younger adults and pooled subject groups across both step directions and for both task conditions are shown in Table 3. For the pooled populations, ICC values were good to excellent (0.74–0.92, p < 0.001, Table 3) with the exception of the swing phase (0.48 and 0.54 for single and dual task conditions, respectively). For elderly individuals the ICC values were good to excellent, ranged from 0.74–0.88 for single task and somewhat lower 0.62–0.85 for dual task (Table 3), but for the swing phase of both single and dual tasks where values were poor although statistically significant (0.47 and 0.42, respectively, p < 0.05, Table 3). For the adult group ICC's were excellent (0.76–0.93, p < 0.000, Table 3) except for the swing phase under single and dual task conditions (0.41, p = 0.006 and 0.54, p < 0.000, respectively).

**Table 3:** ICC values for intratester reliability

		**IPD**	**PPD**	**SPD**	**FOT**	**FCT**
**Single Task**	Elderly N = 16	0.77***	0.83***	0.47*	0.88***	0.74***
	Younger N = 22	0.86***	0.88***	0.41**	0.91***	0.76***
	Pooled N = 38	0.86***	0.86***	0.48***	0.91***	0.79***
**Dual Task**	Elderly N = 16	0.73***	0.62**	0.42*	0.85***	0.71***
	Younger N = 22	0.84***	0.79***	0.54***	0.93***	0.85***
	Pooled N = 38	0.86***	0.74***	0.54***	0.92***	0.86***

Table 3 shows ICC(2,1) values and significance levels (in parenthesis) for intratester reliability (Rater 1 test #1 vs. Rater 1 test #2) for step execution parameters Initiation Phase Duration (IPD), Preparatory Phase Duration (PPD), Swing Phase Duration (SPD), Foot-Off Time (FOT) and Foot-Contact Time (FCT) under single and dual task conditions calculated separately for elderly subjects (N = 16), younger adult subjects (N = 22) and the pooled population of elderly and younger adult subjects (N = 38). An average of all six step trials (three forward and three backward) was used in the analysis.

As for intratester reliability, foot-off time showed the highest and most consistent ICC values for elderly persons, under both single and dual task conditions (0.88 and 0.85, p < 0.000, respectively) as well as the highest internal consistency (Cronbach's alpha, 0.92 and 0.94, respectively). The lowest values were found for swing phase duration (Cronbach's alpha, between 0.59 and 0.64).

### Effect of trial repetition

**Figure 2:**
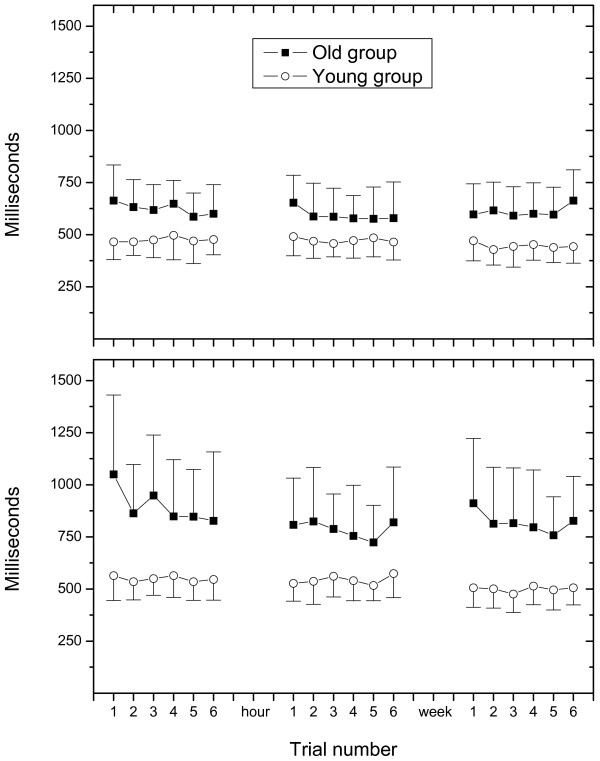
Foot-off times from the 1^st^, 2^nd ^(an hour later) and 3^rd ^(a week later) test session for the elderly and younger group of subjects, respectively, across 6 trials and Task conditions: single task condition (top) and dual task condition (bottom). Values represent mean foot-off times of elderly (filled square) and young (open circle) subjects. Vertical bars indicate one standard deviation. Notice decrease in foot-off times during the first test session for the elderly subjects, especially between the first and second trial under dual task conditions. A similar decrease is seen under dual task conditions for the third test session. Such effects were not present in the younger group.

Figure 2 shows average foot-off time for single and dual task conditions for both populations of subjects across all three test session. Overall, ICC's for foot-off time were highest across all parameters and conditions and were excellent (0.79–0.93), apart from 0.68 in dual task stepping for the younger group. During the 1^st ^test session there was a non-significant (p = 0.12) decrease in the average dual task foot-off time for elderly subjects from 1050 ms for the 1^st ^trial to 827 ms for the 6^th ^trial with the largest change seen between the 1^st ^and the 2^nd ^trial (Figure 2). An hour later during the 2^nd ^test session no learning period observed, the average foot-off time for the 1^st ^trial was 808 ms and 820 ms for the 6^th ^trial. A week later, during the 3^rd ^session the foot-off time for the 1^st ^trial was 911 ms and decreased to 827 ms (p = 0.15) for the 6^th ^trial. For the younger group the foot-off time under dual task condition was approximately 500–550 ms across all trials with no noticeable decrease in duration across trials. No significant changes in foot-off time were seen during single task stepping for either age group.

## Discussion

Results from the current study provide show that Step Initiation Phase duration, Time to Foot-off, Time to Foot-contact, Preparatory Phase extracted from the ground reaction force during a voluntary step execution have good to high intra- as well as inter-tester reliability across age groups under both single and dual task conditions. Furthermore, ICC values were independent of step direction allowing pooling of these data. For the elderly population, foot-off and foot-contact time ICC's were good to excellent under both single and dual task conditions (0.68–0.93). The lowest ICC values for the elderly group were seen for the swing phase (0.32–0.77). Previous studies have demonstrated that voluntary step execution is sensitive to the effects of age [8,24] and diseases including hemiparesis [27] and Parkinson's disease [28,29]. Consequently, parameters of a simple voluntary step execution test may be a useful and reliable clinical test measure of a functional task that involves a requirement of balance.

The magnitude of reliability we found was equal to or higher than Intraclass correlations coefficients (ICCs) of physical performance and physiologic assessments reported by Wolinsky's et al. [30]. They tested eighty subjects aged 50 to 65 between 5 and 45 days after a baseline test and found ICC's of 0.81 for grip strength, 0.72 for chair stands, 0.56 for gait speed, 0.60 for one-leg stand, 0.52 for semitandem stand, 0.58 for tandem stand with eyes closed, and 0.27 for tandem stand with eyes open. More recently, Curb et al. [31] found good reliability for one-leg stance (0.69), 10-foot walk (0.59) and Rapid 10-foot walk (0.57) in a sample of 203 Japanese Americans aged 35 to 55 and 56 to 71 years old and without significant functional impairments. They also found high reliability for the 6-minute walk (0.90) and timed chair stands (0.80 for 5 stands and 0.84 for 10 stands) Sherrington and Lord [32] investigated the test-retest reliability of measures of strength, balance, gait and functional performance in 30 elderly subjects following hip fracture. They found high ICC (3,1) values for hip abduction strength (0.75–0.86), hip flexor strength (0.66–0.80) and knee extensor strength (0.68–0.94). ICCs for postural sway measures ranged 0.59–0.89 for single leg stance. For the step test they found ICC (3,1) values of 0.85–0.92 very similar to values for foot-off and foot-contact times (ICC (2,1) seen in current study. Wolinsky's et al. [30] found that physiologic assessments including systolic and diastolic blood pressure, height, weight, body fat, and peak expiratory flow had ICCs > 0.89, Except for blood pressure (ICC's of 0.51 and 0.55 for systolic and diastolic).

In general, the lowest ICC values across all conditions were found for the swing phase parameter. The most likely explanation for this discrepancy relates to the instructions provided to the subjects before the test. The only restriction given was to step outside the force platform they were standing on. No specific instruction regarding step length was provided, which may have caused within as well as between subject variability that could result in lower ICC's. Consequently, it is reasonable to assume that more precise instructions to the subject regarding step length, e.g. requiring the foot to land between two lines, would increase the reliability of the swing phase parameter.

## Conclusion

The present study has shown that voluntary step can provide highly reliable test parameters in healthy adult and elderly individuals. The test was reliable under both single and dual task conditions especially for foot-off and foot contact times, which is of importance for clinicians to know since differences between younger and elderly individuals is far more pronounced under dual task conditions [8]. This would indicate that the dual task test may be useful as a prospective screener of individuals at risk of falling. The test was consistent over repeated applications of the measurement procedure and can be used to evaluate the performance of four different events: step initiation and preparation phases as well as foot-off and foot-contact times. We suggest that six trials should be administered, three forward and three backwards in random order and that an average of all six trials should be used as an indicator of performance. To stabilize the response, mainly in elderly individuals, at least three learning trials should be administered prior to testing commences.

## Competing interests

The author(s) declare that they have no competing interests.

## Authors' contributions

IM was involved in planning and conducting experiments as well as data analysis and interpretation and drafting of the manuscript. NR and IS conducted the tests and were involved in experimental planning. LO was involved in experimental design, data analysis and interpretation as well as drafting and revising of the manuscript. IM and LO have both given final approval of the current manuscript.
